# Development and evaluation of user-tested Thai patient information leaflets for non-steroidal anti-inflammatory drugs: Effect on patients’ knowledge

**DOI:** 10.1371/journal.pone.0210395

**Published:** 2019-01-09

**Authors:** Narumol Jarernsiripornkul, Pacharaporn Phueanpinit, Juraporn Pongwecharak, Janet Krska

**Affiliations:** 1 Faculty of Pharmaceutical Sciences, Khon Kaen University, Khon Kaen, Thailand; 2 Faculty of Pharmacy, Thammasat University, Pathumthani, Thailand; 3 Medway School of Pharmacy, Universities of Greenwich and Kent, Kent, United Kingdom; Universiteit van Amsterdam, NETHERLANDS

## Abstract

**Introduction:**

Thai patients do not routinely receive patient information leaflets (PILs) with medicines, so awareness of safety issues is low. This study aimed: i) to develop Thai PILs for NSAIDs and subject these to user-testing, and ii) to assess the potential value of PILs from the patient perspective and effect on patient knowledge.

**Methods:**

Four PILs for NSAIDs were developed and subjected to multiple rounds of user-testing by the general public. Self-administered questionnaires were distributed to orthopaedic out-patients prescribed one of these NSAIDs, assessing knowledge before and after providing a PIL. The follow-up questionnaire also sought use of and views on the PILs using a visual analogue scale (VAS).

**Results:**

1,240 baseline questionnaires were completed; only 13.5% of patients had good knowledge. 688 patients returned follow-up questionnaires (55.5%), of whom75% had good knowledge. In patients completing both questionnaires, mean knowledge score increased from 6.22±1.40 to 8.42±1.41 (p<0.001). Patients with high educational levels had high baseline scores (OR = 2.728) and showed greatest improvement in knowledge (OR = 5.628). 90% (625) of follow-up respondents indicated they read all information in the PILs. All also agreed that these PILs should distributed to all patients taking NSAIDs. The median VAS score for usefulness was 9.3 (IQR 8.6–10.0).

**Conclusions:**

User-testing of PILs was feasible in a Thai population and enabled the development of acceptable and desirable PILs. PILs could improve patients' knowledge about their medicine, particularly among those with higher educational level. User-tested PILS could meet the need for more written medicine information.

## Introduction

Written medicine information is a widely accepted essential requirement for supporting patients to gain optimum benefit and reduce harms from the medicines they use [1]. The fact that many patients do not use medication as prescribed [2] has been linked to insufficient medicines information in general [3], and poor communication with healthcare professionals [4], as well as to healthcare professionals’ inadequate knowledge of medicines [5], and limited time for providing information [6].

Patients generally desire more information regarding their medication [7, 8]. Patient information leaflets (PILs) supplied with medicines can increase patients’ knowledge about their medicines [9,10], increase safe use of medicines [11, 12] and act as a reference source, enabling patients to make informed decisions about using medicines, so contributing to patient-centred care [7, 11]. However, some healthcare professionals have concerns that reading PILs may lead to anxiety and non-adherence, despite studies showing that the majority of patients are not so affected [13, 14], and instead are more satisfied if they receive leaflets with risk information than if they do not [15]. PILs to accompany medicines have been developed in many countries, following requirements from governments and other agencies and key principles on the format of written consumer information have been published [16]. Europe, Australia and the United States also promote or require the distribution of PILs with medicines. European regulations clearly state that PILs must be prepared by pharmaceutical companies, in a practical format that people can easily understand [17, 18]. The acceptability and ease of use of written information should be assessed before distribution to patients [11]. Thus, manufacturers of all newly registered medicines are required to assess patients' ability to find and understand information in leaflets, through a process called “User-testing”, which is regarded as so important that products without successful results in this test cannot be marketed in Europe [11, 19]. While there is increasing international effort to improve the quality of medicine information targeted towards patients [20–22], for example by adding a headline section [23] or benefit information [24], the situation in Thailand is very different.

Guidelines for preparing PILs were introduced in Thailand in 2013 [25], however a key difference is that EU requires user-tested PILs to be produced prior to granting marketing authorization, while PILs for medicines normally prescribed by a doctor are still voluntary in Thailand. Moreover the degree of verification of information leaflets, including enforcement of preparing PILs is very limited, in contrast to many countries. The Thai guidelines relating to PILs differ from EU guidelines in the main headings required, they also specify more detail of the content to be included under these headings and require specific formatting. Although the guideline advocates user-testing, no previous study has reported findings from user-testing carried out in Thailand.

Our studies involving Thai patients using non-steroidal anti-inflammatory drugs (NSAIDs) show that they desire more information about their medicines [26, 27]. They perceive risks associated with NSAIDs as low and lack knowledge about potential factors increasing these risks, hence there is a need to improve awareness [28]. Although some health professionals in Thailand do have concerns about the possibility of PILs creating anxiety and non-adherence, the majority agree that PILs should be widely available [29, 30]. In practice, a limited number of leaflets are available, which are often targeted towards prescribers, rather than being specifically designed for patient use; they may be incomplete or provide information of a poor standard [30]. This is particularly true of medicines produced by local pharmaceutical companies. Similar problems have also been found in other countries [18, 31–33].

We therefore set out to develop PILs for selected NSAIDs in Thai language, subject them to user-testing and then assess their potential value from the perspective of patients using these drugs. NSAIDs are widely used in Thailand, being easily obtained without prescription, and adverse drug reactions from NSAIDs are regularly among the most frequently reported to the Thai Food and Drug Administration (FDA) (1984–2017) [34]. The aims of the study were: i) to develop Thai PILs for NSAIDs and subject these to user-testing, and ii) to assess the potential value of PILs from the patient perspective and how they affect patient knowledge about NSAIDs.

## Materials and methods

### Design and setting

The study was approved by the Ethics Committee for Human research, Khon Kaen University [Identifier: HE551130] and conducted in orthopaedic patients at an 800-bed university teaching hospital in the north-eastern region of Thailand. The most frequently prescribed NSAIDs for managing orthopaedic conditions in this hospital (December 2014 to November 2015) were naproxen, diclofenac, celecoxib and etoricoxib. Stage 1 involved development and user-testing of PILs for these four NSAIDs and Stage 2 an evaluation of the PILs developed. Stage 1 was carried out with members of the general public aged attending primary care units and Stage 2 with out-patients prescribed one of the four NSAIDs.

### Development of patient information leaflets

#### Stage 1 development of patient information leaflets

A PIL was developed for each of the four commonly prescribed NSAIDs based on the guideline for leaflet development from FDA Thailand [25], which describes the format, layout and content required. Fourteen trusted sources were used to select key items of importance to patients (information leaflets for healthcare professionals in Thailand, The electronic Medicines Compendium (eMC), The European Medicines Agency (EMA), The Medicines and Healthcare Products Regulatory Agency (MHRA) in the UK, the United States Food and Drug Administration, The Therapeutic Goods Administration (TGA), Medscape, Micromedex, information from the registration of the medicinal formula in Thailand, http://www.patient.co.uk/, The National Institutes of Health (NIH), Australian medicine information, Health Canada (www.hc-sc.gc.ca), and some models of NSAID PILs from FDA Thailand). Any content which was mentioned by at least five of these sources, therefore judged to be of importance to patients, was incorporated into the Thai PILs. The PILs used the six main headings required by the Thai FDA guideline; (1) What is this drug, (2) What you should know before taking, (3) How to use this drug, (4) Things you should do during treatment, (5) Possible harmful effects, and (6) Storage. The formatting also followed the Thai FDA guideline, therefore the PILs were produced onA4 paper, in three columns of text in landscape orientation, with main topics centrally justified in Tahoma bold 14-point and general contents in regular 11-point. The six main headings were highlighted using gray colour. Two pharmacist experts discussed and revised the content of information in PILs prior to user-testing.

#### Stage 2 user-testing

User-testing was conducted following the European Commission (EC) guideline [35] and Thai FDA guideline [25]. The latter requires 12 to 15 questions covering facts, actions and explanations and 10 to 11 users per round. For acceptance, 80% of users should find and understand information in the PIL (90% must be able to find information and 90% of these must understand it). Revision of any weak point should be undertaken followed by repeat testing and, if successful, a final test to confirm this. The general public who were visiting two major primary healthcare units in an urban area in Khon Kaen province, the second largest province in the Northeast region, were invited to participate in this testing. Potential participants had to be aged over 18, with education level no higher than secondary school and verbally indicated no prior use of NSAIDs. People who met these criteria were given a short article to read and their general reading skill judged by their ability to explain the story in the article. Those who were able to complete this assessment were included in user-testing.

Participants were given one of the developed PILs to read, followed by a series of 14 questions to answer after they read the PIL completely. The time taken for finding details of the answers to each question was recorded, as well as participants’ behaviour and any problems encountered during testing were observed. The results from 10 participants were summarized, then the content of the PILs was revised if the results showed it had not passed the test criterion laid down by the Thai FDA. Ten different people completed each round of user-testing, each evaluated only one PIL and three rounds of user-testing were conducted for each PIL. The second testing was conducted for leaflets which did not achieve the criterion, and the third testing (final test) was conducted to confirm repeatability of the results from the second test. The same 14 questions, consisting of a mixture of facts, explanation, and actions, covering all six sections of the PILs were used in each evaluation round (Box 1).

**Box 1. A total of 14 questions used in user-testing**

1. What is the name of this drug? (FACT)
2. What is this drug used for? (FACT)
3. What patients have contraindications to taking this drug? (FACT)
4. What cardiovascular disease risks should you be aware of before you take this drug? (FACT)
5. What drugs do you need to be careful of using in combination with this drug? (FACT)
6. How should you take this drug? (FACT)
7. Why should you take this drug with meals or milk, and drink plenty of water? (EXPLAIN)
8. What should you do if you forget a dose? (ACT)
9. What special cautions should you take while using this drug? (FACT)
10. Can you drink alcohol or beer while taking this drug, and why? (EXPLAIN)
11. What should you do if you need to take vitamins, nutritional supplements, and herbal products while using this drug? (ACT)
12. What side effects can this drug cause? (FACT)
13. If you have severe pain, black or bloody stool, or vomiting blood while using this drug, what should you do? (ACT)
14. How should you store this drug? (FACT)

#### Stage 3 potential value of PILs

Eligible patients were those prescribed one of the four NSAIDs for at least one month during January to May 2016, identified through screening of medical records at orthopaedic out-patient clinics. At each study visit, all patients with a current prescription for one of the four NSAIDs were invited to participate. Patients below 18 years old, unable to complete the questionnaire and read the PIL themselves or with support, were excluded.

Questionnaires were developed for self-administration at baseline and one month after receiving a PIL. These were assessed for content validity by three pharmacists, the calculated index of consistency was 0.98, following which pilot testing was carried out with ten patients using one of the four NSAIDs to ensure the questionnaire was easy to read and understand. To assess patients’ NSAID knowledge, ten multiple-choice questions, based on the PIL content, were used on both occasions. The baseline questionnaire also included demographic questions (sex, age, educational level, occupations, health insurance, underlying disease, concomitant drug, social history, and history of allergy), while the follow-up questionnaire determined respondents’ use of PILs (sections read and when, frequency of reading), additional sources of information accessed and opinions on future use of this PIL. Visual analogue scales (VAS), ranging from 0 to 10, were used to rate the PIL usefulness (0 = no usefulness, 10 = highest usefulness) and respondents’ anxiety (0 = no anxiety, 10 = highest anxiety) after reading the PIL.

Baseline questionnaires were directly distributed to all patients who agreed to take part, for completion while in the waiting room. All patients who returned the completed baseline questionnaire to the researcher were then given the relevant PIL and follow-up questionnaire, together with a stamped reply envelope, and requested to read the PIL and return completed questionnaires by mail after one month. One follow-up letter and personal contact by phone were used to remind non-responders.

### Sample size

There was no previous study in Thailand which had determined the effects of PILs on knowledge and specifically developed PILs which had been subjected to user-testing in the country had limited availability, therefore we conservatively assumed we chose to use a moderate effect size of 0.25. The sample size for the study was therefore calculated using an alpha error at 0.05, a power of 0.80 (beta = 0.02), and an effect size of 0.25. The approximate sample size required was 130 cases for each NSAID, a total of 520. Based on the response rate from a previous study in patients taking prescribed NSAIDs in this hospital of 42% [36], the number of patients required was therefore 1,240.

### Statistical analysis

Data from the baseline and follow-up questionnaires were analyzed using IBM SPSS for Windows (version 19.0). Simple descriptive statistics were used to describe respondent characteristics, frequency of correct responses to each knowledge question and patients’ use of the PILs. Scores for perceived benefits of PILs and anxiety level after reading are presented as median with interquartile range (IQR). The ten NSAID knowledge questions were each given a score of one, hence the maximum score was ten. The summed scores were divided into two levels; good knowledge (score 8 or over), and less than good knowledge (score less than 8), to enable factors associated with baseline knowledge level to be determined.

Chi-square test and Fisher’s exact test were used to compare categorical data between responders to the follow-up questionnaire and non-responders. Independent t-test was used to compare the means of knowledge score at baseline. McNemar’s test was used to compare scores for individual items between baseline and follow-up. Paired samples test was used to compare knowledge scores at baseline and follow-up. Demographic characteristics found in univariate analyses to be significantly associated with baseline NSAID knowledge score and the change in total scores were included in multivariate analyses using linear regression.

## Results

### User-testing

A total of 240people participated in user-testing, 90 people in first round testing (9 developed PILs were tested), 90 people in second round (3 PILs were confirmed and 6 PILs were re-tested), and 60 people in third round (6 PILs were confirmed).From the first round of user-testing, observations noted that some participants had problems finding answers for questions concerning contraindications and concomitant drugs (Questions 3 and 5). It was noted that they frequently did not notice the main topics of information in PILs, thus key topics and clear words at the beginning of sentences appeared very important, affecting ability to find answers to the questions. Difficulties were observed when the words at the beginning of the target subtopics did not provide the answers. For example, in relation to concomitant drugs (Question 5), the answer was located under the heading “Precautions when using this drug” in topic 4, beginning: “Patients who are taking…”. Therefore, the wording was subsequently revised to: “Drugs you must be careful about when taking this drug…”. Additionally, some participants did not understand the difference between "Who should not use this drug?" and "Precautions when using this drug". This meant the question on contraindications (Question 3) was frequently not answered correctly, with respondents giving answers relating instead to precautions. Finally, the sub-topic heading: "When should you not use this drug?" was changed to "Who should not use this drug?" for the second and final rounds of testing. Moreover, several revisions were made to the formatting after the first round of testing: a text box with white background was used to draw attention to subtopics, and text size was increased to 18-point bold for the drug name, 16-point bold for six main topics, 12-point bold for subtopics and 11.5-point for general contents. After revision, all four PILs passed the criteria in the second round and a final test was then conducted to ensure a consistent result.

### Response rates and demographic details

A total of 1,274 patients were verbally invited by the researcher, however, 34 patients declined therefore 1,240 patients were willing to engage in the study. A total of 1,240 patients completed baseline questionnaires, 688 (55.5%) of whom returned completed follow-up questionnaires. Of the latter, 160 questionnaires were returned from naproxen users (51.6%), 168 from diclofenac users (54.2%), 179 from celecoxib users (57.7%), and 181 from etoricoxib users (58.4%).

The characteristics of responders and non-responders (Table 1) are presented that approximately two-thirds of the total 1,240 respondents were female (N = 837, 67.5%), the average age was 54.83±12.58 years (minimum-maximum age, 18–77 and nearly half had educational level above high school (N = 522, 42.1%). Over a third (N = 472, 38.1%) had underlying diseases, most commonly hypertension (N = 263, 21.2%), diabetes mellitus (N = 122, 9.8%), and dyslipidemia (N = 79, 6.4%) and almost all patients were using one or more concomitant drugs (1181; 95.2%). In addition, approximately half had used a prescription NSAID for more than three months (N = 588, 47.4%). Slightly more civil servants, those using the Civil Service benefits scheme smokers, people using herbal medicines and those who had used NSAIDs for over three months returned follow-up questionnaires.

**Table 1:** Characteristics of patients completing both questionnaires, compared to non-respondents.

Demographic data	Number of patients (%)			*p*-value
	Non-respondents (N = 552)	Respondents (N = 688)	Total (N = 1,240)	
**Gender**				
Male	168 (30.4)	235 (34.2)	403 (32.5)	0.180^b^
Female	384 (69.6)	453 (65.8)	837 (67.5)	
**Age**				
Mean±S.D.	54.49±12.81	55.11±12.40	54.83±12.58	0.389^c^
Median (IQR)	57 (49–64)	57 (50–64)	57 (49–64)	
**Educational level**				
≤ High School	345 (62.5)	373 (54.2)	718 (57.9)	**0.003**^**a**^
> High School	207 (37.5)	315 (45.8)	522 (42.1)	
**Occupations**				
None	202 (36.6)	230 (33.4)	432 (34.8)	**<0.001**^**a**^
Civil servant	111 (20.1)	218 (31.7)	329 (26.5)	
Farmer	112 (20.3)	124 (18.0)	236 (19.0)	
Other	127 (23.0)	116 (16.9)	243 (19.6)	
**Health Insurance**				
UC Gold card^d^	185 (33.5)	166 (24.1)	351 (28.3)	**0.001**^**a**^
CSMBS^e^	341 (61.8)	482 (70.1)	823 (66.4)	
Other	26 (4.7)	40 (5.8)	66 (5.3)	
**Underlying disease**				
Have	213 (38.6)	259 (37.7)	472 (38.1)	0.593^a^
- Hypertension	.1)	141 (20.5)	263 (21.2)	
- Diabetes mellitus	58 10.5)	64 (9.3)	122 (9.8)	
- Dyslipidemia	37 (6.7)	42 (6.1)	79 (6.4)	
- Peptic ulcer/GERDs	11 (2.0)	31 (4.5)	42 (3.4)	
- Asthma	9 (1.6)	16 (2.3)	25 (2.0)	
- CVS disease	8 (1.45)	11 (1.6)	19 (1.5)	
- Thyroid	16 (2.9)	8 (1.2)	24 (1.9)	
- Kidney disease	0 (0)	1 (0.2)	1 (0.1)	
**Drinking**	43 (7.8)	76 (11.1)	119 (9.6)	0.065^b^
**Smoking**	9 (1.6)	25 (3.6)	34 (2.7)	**0.036**^**b**^
**Have drug allergy history**	18 (3.3)	29 (4.2)	47 (3.8)	0.455^b^
**Have concomitant drugs**	523 (94.7)	658 (95.6)	1181 (95.2)	0.503^b^
**Taking herbs and supplement**	16 (2.9)	55 (8.0)	71 (5.7)	**<0.001**^**b**^
**Duration of NSAID use**				
≤ 3 months	305 (55.3)	347 (50.4)	652 (52.6)	**0.018**^a^
4–12 months	208 (37.7)	261 (37.9)	469 (37.8)	
>12 months	39 (7.1)	80 (11.6)	119 (9.6)	
**Type of NSAID use**				
Non-selective NSAIDs	292 (52.9)	328 (47.7)	620 (50.0)	0.067^a^
Selective COX-2 NSAIDs	260 (47.1)	360 (52.3)	620 (50.0)	

^a^ Chi-square test

^b^ Fishers’ Exact test

^c^ Independent t-test

^d^ The UC (Universal Coverage) Gold Card

^e^ The Civil Servant Medical Benefits Scheme (CSMBS)

Educational levels differed with occupation; over 80% of patients with no occupation (82.9%) and farmer (97.9%) had only high school level education, whereas 98% of civil servants had higher education level. Most of the civil servants (90%) were able to access CSMBS while only 60% of farmers were able to use this scheme and the remaining 40% used universal health coverage.

### Accuracy of NSAID knowledge

The proportion of patients with a baseline total score ≥ 8 points was 13.5% (N = 168/1240), with no difference between those who returned follow-up questionnaires and those who did not (6.18±1.25 Vs 6.22±1.40, p = 0.673). Of the 688 completing follow-up questionnaires, 519 (75.4%) achieved a score of 8 or more and mean NSAID knowledge score changed from 6.22±1.40 at baseline, to 8.42±1.41 at follow-up (paired t-test; t = -38.64, df = 687, *p*<0.001). Users of non-selective NSAIDs had slightly lower scores than selective NSAID users at baseline (6.07±1.51 Vs 6.35±1.27, p<0.05), whereas there was no significant difference in scores at follow-up between these two groups.

At baseline, the majority of respondents gave incorrect answers to questions covering risk factors for using NSAIDs (N = 572, 83.1%), and the effect of drinking alcohol while using NSAIDs (N = 538, 78.2%), while many gave incorrect responses to questions about drug-drug interactions (N = 369, 53.6%), side effects (N = 347, 50.4%), symptoms requiring stopping the drug (N = 303, 44.0%) and missed-dose management (N = 245, 35.6%) (Table 2). At follow-up, the proportion of respondents answering all questions correctly increased significantly, with only two questions, both having high baseline correct responses, showing no increase.

**Table 2:** Frequency of correct responses to NSAID knowledge questions at baseline and follow-up.

Knowledge items	No. of patients (%)		*p*-value^a^
	Pre-test	Post-test	
**Q1**: Indication of NSAIDs			
Correct answer	654 (95.06)	686 (99.71)	**<0.001**
Incorrect answer	34 (4.94)	2 (0.29)	
**Q2**: Contraindication of NSAIDs			
Correct answer	524 (76.16)	678 (98.55)	**<0.001**
Incorrect answer	164 (23.84)	10 (1.45)	
**Q3**: Factors increasing risk of taking NSAIDs			
Correct answer	116 (16.86)	342 (49.71)	**<0.001**
Incorrect answer	572 (83.14)	346 (50.29)	
**Q4**: Side effect of NSAIDs			
Correct answer	341 (49.56)	576 (83.72)	**<0.001**
Incorrect answer	347 (50.44)	112 (16.28)	
**Q5**: Reason for taking after meals			
Correct answer	649 (94.33)	679 (98.69)	**<0.001**
Incorrect answer	39 (5.67)	9 (1.31)	
**Q6**: Management of missed dose			
Correct answer	443 (64.39)	437 (63.52)	0.742
Incorrect answer	245 (35.61)	251 (36.48)	
**Q7**: Things to do while taking NSAIDs			
Correct answer	683 (99.27)	682 (99.13)	1.000
Incorrect answer	5 (0.73)	6 (0.87)	
**Q8**: The effect of drinking alcohol			
Correct answer	150 (21.80)	509 (73.98)	**<0.001**
Incorrect answer	538 (78.20)	179 (26.02)	
**Q9**: Symptoms requiring stopping NSAID and going to doctor			
Correct answer	385 (55.96)	574 (83.43)	**<0.001**
Incorrect answer	303 (44.04)	114 (16.57)	
**Q10**: Drug interactions			
Correct answer	319 (46.37)	611 (88.81)	**<0.001**
Incorrect answer	369 (53.63)	77 (11.19)	
**Total knowledge score**			
Pre-test; mean±S.D.	6.22±1.40	**<0.001**^**b**^	
Post-test; mean±S.D.	8.42±1.41		
Mean difference	2.20±1.49		

^a^ McNemar test

^b^ Paired t-test

Factors associated with NSAID knowledge

Linear regression analysis (Table 3) found that higher baseline knowledge scores were significantly associated with being female, having a higher educational level, and using selective COX-2 NSAIDs. However, baseline knowledge scores were significantly lower in patients with older age.

**Table 3:** Adjusted linear regression for knowledge score in patients at baseline.

Factors	b	SE_b_	****β****	t	95% CI		*p*-value
					Lower	Upper	
Gender	0.231	0.078	0.081	2.954	0.077	0.384	**0.003**
Age	-0.016	0.003	-0.154	-4.844	-0.023	-0.010	**<0.001**
Educational level	0.654	0.080	0.242	8.159	0.497	0.812	**<0.001**
Underlying disease	-0.117	0.076	-0.043	-1.542	-0.265	0.032	0.123
Type of NSAID use	0.271	0.078	0.102	3.488	0.119	0.423	**0.001**
	Constant 6.576; SE_est_ = ±0.191						
	R = 0.123; Adjusted R^2^ = 0.120; F = 34.767; *p*-value<0.001						

*b* denotes the variable estimate

*SE*_*b*_ denotes the standard error of the variable estimate

*β* denotes the standardized estimate

*t* denotes the t-value

Adjusted for gender, age, educational level, health insurance, occupations, underlying disease,number of disease, concomitant drugs, and type of NSAID use

The change in knowledge score following provision of the PILs was assessed using multiple linear regression (Table 4). Educational knowledge had the greatest impact on improving knowledge, whereas patients taking selective COX-2 NSAIDs, using the Civil Servant Medical Benefits Scheme (CSMBS) health insurance, and those with a history of drug allergy were less likely to increase their knowledge score.

**Table 4:** Adjusted linear regression for the change in knowledge score.

Factors	b	SE_b_	****β****	t	95% CI		*p*-value
					Lower	Upper	
Educational level	0.658	0.115	0.219	5.698	0.431	0.884	**<0.001**
Type of NSAID use	-0.393	0.120	-0.132	-3.285	-0.628	-0.158	**0.001**
Health insurance	-0.284	0.120	-0.098	-2.368	-0.520	-0.049	**0.018**
History of drug allergy	-0.613	0.274	-0.082	-2.236	-1.151	-0.075	**0.026**
	Constant 2.365; SE_est_ = ±0.110						
	R = 0.072; Adjusted R^2^ = 0.066; F = 13.166; *p*-value<0.001						

*b* denotes the variable estimate

*SE*_*b*_ denotes the standard error of the variable estimate

*β* denotes the standardized estimate

*t* denotes the t-value

Adjusted for gender, age, educational level, health insurance, occupations, number of disease, drinking, history of drug allergy, and type of NSAID use.

#### Self-reported use of and views on the developed PILs

Approximately 90% of follow-up respondents indicated they read all information in the PILs (N = 625), while the remainder (N = 63) read some sections, in particular: what is the drug for (81%), administration (82%), and side effects (36%). Moreover, 83.4% of respondents claimed they read the PIL shortly after receiving it (N = 574), while 16.1% only read it when they had doubts about this drug (N = 111). The median VAS score for usefulness from the 688 respondents was 9.3 (IQR 8.6–10.0), with only three scoring usefulness below 5.0. The median score for anxiety was 2.9 (1.1–5.0), although six respondents gave a score of 10.0(Table 5). There were no statistically significant differences between frequency of reading and demographic data, however, we found a significant association in usefulness and anxiety score after reading the developed PILs. Patients with education level above high school tended to provide higher usefulness scores than patients with high school level and lower (9.31±0.915 vs 8.82±1.273, *P*<0.001). Moreover, selective NSAID users were more likely to provide higher anxiety scores than non-selective NSAID users (3.69±2.181 vs 2.74±2.533, *P*<0.001).

**Table 5:** Mean and median score for frequency of reading PILs, usefulness and anxiety after reading.

Items	Mean±S.D.	Median (IQR)	Min-Max
Frequency of reading in one month (time)	1.95±1.25	2 (1–2)	1–16
Usefulness of PILs (VAS score)	9.04±1.15	9.3(8.6–10.0)	1.0–10.0
Anxiety after reading (VAS score)	3.38±4.66	2.9(1.1–5.0)	1.0–10.0

*IQR* denotes the interquartile range

*VAS* denotes visual analog scale

All 688 patients also agreed that these PILs should distributed to patients taking NSAIDs, especially with their first prescription (N = 457, 66.5%) and on request (N = 276, 40.2%). Furthermore, 38.1%of patients (N = 262) indicated the information they contain should be made publicly available on a general website for patients. Only 183 respondents (26.6%) reported they received drug information from additional sources during this study, mostly from a pharmacist (N = 136, 74.3%), a doctor (N = 59, 32.2%) or the internet (N = 27, 14.7%).

## Discussion

The processes of developing and user-testing of the PILs undertaken in this study is of particular importance as it was carried out in a developing country using the local language. The Thai population have previously been shown to have limited knowledge of medicines and limited access to documented medicine information [28, 30], hence the participants involved in user-testing were unfamiliar with PILs. The process of user-testing identified potential problems with PILs, requiring changes which meant that the final PILs distributed to patients differed from the Thai FDA guideline requirements in some respects, although it retained the six required headings. This illustrates the need to conduct user-testing and to involve patients in the development of information leaflets, as health professionals and manufacturers may not anticipate problems which are likely to occur in practice. The initial words in each sentence were particularly important to enable participants to find information. We also found that the general public did not understand the difference between precautions and contraindications; some of them believed that precautions meant not being allowed to use the medicine. Reports of user-testing are relatively rare outside of developed countries [37], but the process is essential if written medicines information is to meet the needs of local populations.

In addition to user-testing, our study involved a large sample of patients actually using the medicines for which PILs were developed, hence their views on the usefulness of the materials were potentially of greater value than those of user-testers. Most of these considered the user-tested PIL useful and desirable and there was a statistically significant improvement in NSAID knowledge score after receiving the PILs. The method used was similar to that of most studies assessing the impact of PILs on patient knowledge—a unique measure devised for the study, providing correct responses to questions concerning key items of information, with an arbitrary cut-point of 80% indicating ‘good’ knowledge [38]. As there is no universally accepted level which constitutes ‘good’ knowledge, we judged that 80% was sufficient to ensure that patients had a good understanding using to ensure safe use of their NSAID. Although total scores increased, there were still fewer than 50% who were able to correctly answer the question on factors increasing the risks of NSAID use. There is a need for greater appreciation of NSAID risks among patients, both in Thailand and elsewhere [28, 39, 40]. Our study also found no increase in the proportion who responded correctly to the question regarding management of missed doses after being given the PIL. This could suggest that the wording on this topic was inadequate in some way, although it was specifically included in the user-testing.

Patients with educational level beyond high school were most likely to have a good baseline knowledge level and also to improve their knowledge score at follow-up. Moreover, females and patients taking selective COX-2 NSAIDS had higher baseline scores than others, but were less likely to show improvements after receiving the PIL. Both of these groups may have received more information before joining the study, as PILs are more widely available for selective NSAIDs in Thailand [30], and drug allergy cards are provided [41].

Studies in other countries have reported that reading information leaflets increases patients’ knowledge [9, 10, 42–44]. Indeed, a previous study in the United States found that patients receiving information leaflets were 2.78 times more likely to have good knowledge regarding risk information than patients not receiving leaflets [44], while a Turkish study found that both verbal and information leaflets greatly improved knowledge [42]. To our knowledge, only one study has been reported in Thailand, which found that provision of both verbal and written information about drug allergies increased knowledge more than the provision of a drug allergy card alone [41].

There is a clear need and desire for more written medicines information in the form of PILs in Thailand [26, 30]. The concerns identified among some Thai health professionals that PILs may cause anxiety [29, 45] are not supported by evidence from studies elsewhere [13–15] and results from our follow-up questionnaire support this. Thai PILs, similar to those in other countries, could cover all essential issues which patients need to know to ensure safe and optimal use of NSAIDs and are seen as desirable by both patients and health professionals [26, 29, 45]. Although patients with higher educational level are more likely to read PILs [10], have greater baseline knowledge [46], and as this study shows, are more likely to improve their knowledge as a result, PILs were considered useful by the large majority of patients in our study, regardless of educational level. Our study shows that user-tested PILs were not only acceptable to this Thai population, but may also have increased patients’ knowledge about medicines, thus could potentially contribute to safer use of medicines in Thailand.

This study had several limitations, the questions developed for user-testing were not validated or assessed for reliability by experts, but were designed to cover all six sections of the PILs. The response rate of 55.5% to follow-up questionnaires was better than expected, therefore the number of patients who returned these (N = 688) was sufficient to meet the estimated sample size requirement (520 cases). However the study was conducted in only one teaching hospital, hence the results may not be generalizable beyond this population. It is important to recognize that this was a naturalistic study, therefore all patients were able to receive information related to NSAIDs from healthcare professionals as normal, in addition to the PIL provided. In reality, approximately a quarter of participants did obtain information from other sources during the study. More importantly, this study had no control group of patients who did not receive a PIL, therefore, no firm conclusions can be drawn about whether the PILs we provided were responsible for changes in knowledge scores. The post-test knowledge score was obtained using a postal self-completed questionnaire, which respondents could have completed at any time after receiving the PIL. They could also have used the PIL or sought additional information to help them answer the questions. Indeed it is possible that completing the questionnaire itself could have provoked some patients to seek answers to questions they found difficult.

Future work could assess the impact of PILs on retention of knowledge and behaviours in relation to medicines, such as adherence, over longer time periods in Thai populations, using randomized controlled designs. In addition studies are required to determine what patients’ preferences are for written medicine information in Thailand, to enable PILs to more effectively meet their needs. Studies elsewhere suggest that individually tailored information is seen as desirable, however this is likely to be feasible only through the use of sophisticated automated computer systems which can adapt generic information to the needs of an individual [47]. At present even generic information is not widely available in Thai pharmacies hence such development would require considerable investment.

## Conclusions

User-testing of written medicine information in the form of PILs was feasible in a Thai population and resulted in the development of PILs viewed as acceptable and desirable by patients. PILs could improve patients' knowledge about their medicine, particularly among those with higher educational level. While further studies are needed to assess whether PILs can improve patient knowledge in Thailand, carefully drafted user-tested PILS could meet the need for more written medicine information.

## Supporting information

S1 File Pretest questionnaire. Survey of patients’ knowledge of Non-Steroidal Anti-inflammatory Drugs.
(PDF)

S2 File Posttest questionnaire. Survey of patients’ knowledge of Non-Steroidal Anti-inflammatory Drugs.
(PDF)
