# Supplementary material for: Development and evaluation of user-tested Thai patient information leaflets for non-steroidal anti-inflammatory drugs: Effect on patients’ knowledge
Source: PLoS One. 2019 Jan 9;14(1):e0210395. doi: 10.1371/journal.pone.0210395 (PMC6326498; doi:10.1371/journal.pone.0210395)
Supplement: S1 File — Survey of patients’ knowledge of Non-Steroidal Anti-inflammatory Drugs. (PDF) [file pone.0210395.s001.pdf]

Patient Code **G101_N**………

QUESTIONNAIRE:
Survey of patients' knowledge of Non-Steroidal Anti-inflammatory Drugs

---

Part 1 Baseline Information

Explanation: Please tick a box (✓) or answer the following questions about you. Your answers to the survey will be kept confidential and there will be no effect on your treatment.

1. Sex ❒ Male ❒ Female
2. Age …………………… years old
3. Education
   - ❒ Primary school
   - ❒ Secondary school
   - ❒ Senior high school
   - ❒ Diploma
   - ❒ Bachelor's degree
   - ❒ Master's degree
   - ❒ Doctor's degree
   - ❒ Etc. (please specify) …………
4. Occupation
   - ❒ Unemployed
   - ❒ Agriculturist
   - ❒ Self employed business
   - ❒ Work as employee
   - ❒ Student/Academic
   - ❒ Private corporation
   - ❒ Government officer
   - ❒ State enterprise
   - ❒ etc. (please specify) ………………………………
5. What is your health insurance?
   - ❒ The Universal Coverage (Gold Card)
   - ❒ The Civil Servant Medical Benefits Scheme
   - ❒ Social security
   - ❒ Self-pay
   - ❒ Etc. (please specify) ………………………………
6. Do you have underlying disease?
   - ❒ No
   - ❒ Yes (please specify) ………………………………..…………………………………………
7. A history of drinking
   - ❒ No
   - ❒ Yes, average…………… glasses/week
8. A history of smoking
   - ❒ No
   - ❒ Yes, average ……………..roll/day
9. A history of drug allergy
   - ❒ No
   - ❒ Yes (please specify) the number of allergy medication …….. drug

     Drug name/ your allergy symptoms ....
10. Currently, the number of medication that you are taking .... drugs

    (Please list all medications if you remember) ……………………………………………………..
11. Do you take vitamin supplements and herbal medicine?
    - ❒ No
    - ❒ Yes (please specify) ……………………………………………………………………………

Pre-test questionnaire

Part 2 Knowledge and understanding of medicine

Explanation: The following questions will be asked about your understanding about the medicine in the image below (you are currently using). Please tick a box (✓) in the following questions which matches your knowledge and understanding.

Current medicine

Generic name Naproxen

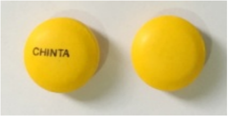

OR

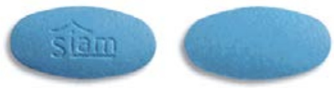

Brand name Synogin
(naproxen 250 mg)

Brand name Annoxen-S
(naproxen 275 mg)

1. What is this medicine used for…….?
   - ❐ Increase bone mass
   - ❐ Improve nervous system
   - ❐ Relief pain and inflammation in bone and muscle diseases
2. Who have contra-indication for use?
   - ❐ Patients with age >40 years
   - ❐ Patients with GI bleeding
   - ❐ Patients with sinusitis
3. Who have risks on gastrointestinal system if using this medicine?
   - ❐ Elderly
   - ❐ Patients with dyslipidemia
   - ❐ Patients with diabetes mellitus
4. What is side effect from using this medicine?
   - ❐ Dry cough
   - ❐ Muscle pain
   - ❐ Heart burn
5. Why should you take this medicine after meals?
   - ❐ To increase drug action
   - ❐ To reduce GI irritation
   - ❐ To avoid forgetting to take medicine
6. What should you do if you miss a dose?
   - ❐ Keep the missing tablet to take with next dose
   - ❐ Take medicine immediately when you recall, with the next dose
   - ❐ Skip this dose and wait for take next dose
7. What should you do while taking this medication?
   - ❐ Drink plenty of water with this medicine
   - ❐ Taking medicine with juice
   - ❐ Bring this medicine to others who have suffered symptoms like you
8. What is the result from drinking alcohol while taking this medicine?
   - ❐ The medicine may have slow action
   - ❐ This medicine is ineffective in treatment
   - ❐ Increase risk of gastrointestinal ulcer
9. What symptoms mean you should stop taking this medicine, and tell doctor immediately?
   - ❐ Flatulence, Indigestion
   - ❐ Red skin, Bruise on skin
   - ❐ Drowsiness, Dizziness

10. If you take this medicine with any other medicine, which may increase the risk of harm?

- ❐ Aspirin
- ❐ Nasal decongestion and runny nose relie
- ❐ Lipid-lowering medicine
