# Supplementary material for: Development and evaluation of user-tested Thai patient information leaflets for non-steroidal anti-inflammatory drugs: Effect on patients’ knowledge
Source: PLoS One. 2019 Jan 9;14(1):e0210395. doi: 10.1371/journal.pone.0210395 (PMC6326498; doi:10.1371/journal.pone.0210395)
Supplement: S2 File — Survey of patients’ knowledge of Non-Steroidal Anti-inflammatory Drugs. (PDF) [file pone.0210395.s002.pdf]

Patient Code **G101_N**………

QUESTIONNAIRE:
Survey of patients' knowledge of Non-Steroidal Anti-inflammatory Drugs

----

Part 1 Knowledge and understanding of medicine

Explanation: The following questions will be asked about your understanding about the medicine in the image below (you are currently using). Please tick a box (✓) in the following questions which matches your knowledge and understanding.

Current medicine

Generic name Naproxen

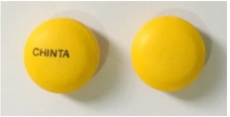

OR

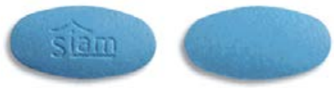

Brand name Synogin
(naproxen 250 mg)

Brand name Annoxen-S
(naproxen 275 mg)

1. What is this medicine used for…….?
   - ❒ Increase bone mass
   - ❒ Improve nervous system
   - ❒ Relief pain and inflammation in bone and muscle diseases
2. Who have contra-indication for use?
   - ❒ Patients with age >40 years
   - ❒ Patients with GI bleeding
   - ❒ Patients with sinusitis
3. Who have risks on gastrointestinal system if using this medicine?
   - ❒ Elderly
   - ❒ Patients with dyslipidemia
   - ❒ Patients with diabetes mellitus
4. What is side effect from using this medicine?
   - ❒ Dry cough
   - ❒ Muscle pain
   - ❒ Heart burn
5. Why should you take this medicine after meals?
   - ❒ To increase drug action
   - ❒ To reduce GI irritation
   - ❒ To avoid forgetting to take medicine
6. What should you do if you miss a dose?
   - ❒ Keep the missing tablet to take with next dose
   - ❒ Take medicine immediately when you recall, with the next dose
   - ❒ Skip this dose and wait for take next dose
7. What should you do while taking this medication?
   - ❒ Drink plenty of water with this medicine
   - ❒ Taking medicine with juice
   - ❒ Bring this medicine to others who have suffered symptoms like you
8. What is the result from drinking alcohol while taking this medicine?
   - ❒ The medicine may have slow action
   - ❒ This medicine is ineffective in treatment
   - ❒ Increase risk of gastrointestinal ulcer

9. What symptoms mean you should stop taking this medicine, and tell doctor immediately?
   - ❒ Flatulence, Indigestion
   - ❒ Red skin, Bruise on skin
   - ❒ Drowsiness, Dizziness

10. If you take this medicine with any other medicine, which may increase the risk of harm?
   - ❒ Aspirin
   - ❒ Nasal decongestion and runny nose relie
   - ❒ Lipid-lowering medicine

Part 2 Questions about the received patient information leaflet

Explanation: Please tick a box (✓) or answer the following questions about you.

1. Do you read the received information leaflet?
   - ❒ Yes, I read all
   - ❒ Yes, I read some parts (Please ✓all parts which you read)
     - ○ What medicine is used for
     - ○ Who should not take medicine
     - ○ Precautions in use
     - ○ Administration
     - ○ When miss dose
     - ○ When take over dose
     - ○ Things you should do while you are taking medicine
     - ○ Side effects
     - ○ Storage
   - ❒ Not read (skip to question 6)

2. When do you read the received leaflet first?
   - ❒ When I receive the medicine
   - ❒ When I have doubt/want to know about this medicine
   - ❒ When I have abnormal symptoms
   - ❒ Other (please specify) ……………………………..…

3. Please specify the frequency of reading the information leaflet during 1 month

   about ....times

4. What is score level of usefulness of the received information leaflet for you?

   (Please "×" on the line below, which scale of 0 to 10 score; 0= least useful, 10= most useful)

   No useful 0 ———— 5 ———— 10 **Most useful**

5. After reading this information leaflet, How anxious do you feel about using this medicine?

   (Please "×" on the line below, which scale of 0 to 10 score)

   No anxiety 0 ———— 5 ———— 10 **Very anxious**

6. In the last month, did you receive medicine information from another source?
   - ❒ No
   - ❒ Yes (Please specify) ....

7. Do you think there should be distribution of information leaflets to patients who using this medicine?
   - ❐ Disagree
   - ❐ Agree, when should patients receive leaflets?… (please specify)
     - ☐ When receiving medicines every time
     - ☐ When receiving medicines at first use
     - ☐ Only providing to high-risk patients
     - ☐ The leaflets should be published on the website
     - ☐ When patients need them
     - ☐ Others (please specify) ....
